# Pressure pain thresholds in chronic migraine associated with hypertension

**DOI:** 10.1186/1129-2377-14-S1-P128

**Published:** 2013-02-21

**Authors:** O Grosu, S Odobescu, L Rotaru, I Moldovanu

**Affiliations:** 1 Institute of Neurology and Neurosurgery, Moldova, Republic of

## Introduction

Studies on pain thresholds in migraine populations are contradictory and have not used algometry.

## Objectives

To evaluate pressure pain thresholds (PPT) in chronic migraine patients associated with arterial hypertension (Mg+AHT) and without hypertension (Mg-AHT).

## Methods

Study consisted of 40 chronic migraine (CM) patients divided in 2 groups: Mg+AHT - 18 pts (mean age 46.19 ± 6.77 years), Mg-AHT- 22 pts (mean age 40.77 ± 12.0 years), and 10 healthy controls (mean age 37.56 ± 10.45 years). PPT were obtained bilaterally by mechanical pressure algometry from 15 anatomic points (ophthalmic nerve, temporalis muscle, median nerve, radial nerve, ulnar nerve, Achilles tendon, thenar eminence, suboccipital muscle insertions, trapezius muscle, supraspinatus muscle, second rib, lateral epicondyle, gluteal, great trochanter, knee) using Somedic algometer (SBMEDIC electronics, Sweden). Pressure algometry was applied three times on the same point and pain threshold was calculated as an average value.

## Results

The mean PPT value for all 15 examined points was higher in Mg+AHT vs. Mg-AHT group (447.36 ± 112.32 vs. 377.67 ± 77.71, p<0.05) but didn’t differ from the control group. In Mg-AHT group PPT was lower compared to Mg+AHT and control group (377.67 ± 77.71 vs. 437.00 ± 81.86, p<0.05). In the Mg+AHT pts PPT was higher than in Mg-AHT group for eight application points (53.3%): radial nerve, ulnar nerve, Achilles tendon, thenar eminence, trapezius muscle, supraspinatus muscle, greater trochanter and gluteal. In Mg- AHT group PPT was lower than healthy controls for seven application points (46.6%): radial nerve, Achilles tendon, thenar eminence, greater trochanter, lateral epicondyle, gluteal and knee.

## Conclusion

The arterial hypertension is associated with the increase of pain pressure thresholds evaluated by algometry in chronic migraine with hypertension patients and interacts with hyperalgesia and allodynia phenomena induced by chronic migraine through central and peripheral sensitization mechanisms.
